# Coumarin carbonic anhydrase inhibitors from natural sources

**DOI:** 10.1080/14756366.2020.1788009

**Published:** 2020-08-11

**Authors:** Claudiu T. Supuran

**Affiliations:** Section of Pharmaceutical and Nutraceutical Sciences, Neurofarba Department, Università degli Studi di Firenze, Florence, Italy

**Keywords:** Carbonic anhydrase, coumarin, natural product, prodrug inhibitor

## Abstract

Coumarins constitute a relatively new class of inhibitors of the zinc enzyme carbonic anhydrase (CA, EC 4.2.1.1), possessing a unique inhibition mechanism, acting as “prodrug inhibitors.” They undergo the hydrolysis of the lactone ring mediated by the esterase activity of CA. The formed 2-hydroxy-cinnamic acids thereafter bind within a very particular part of the enzyme active site, at its entrance, where a high variability of amino acid residues among the different mammalian CA isoforms is present, and where other inhibitors classes were not seen bound earlier. This explains why coumarins are among the most isoform-selective CA inhibitors known to date among the many chemotypes endowed with such biological activity. As coumarins are widespread secondary metabolites in some bacteria, plants, fungi, and ascidians, many such compounds from various natural sources have been investigated for their CA inhibitory properties and for possible biomedical applications, mainly as anticancer agents targeting hypoxic tumours.

## 1. Introduction

**Figure 1.**
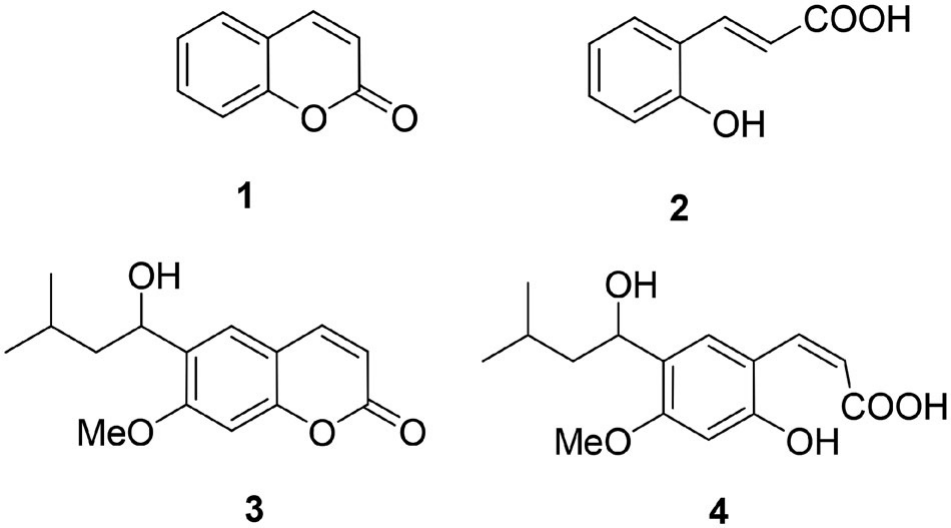
The simplest coumarin, compound **1**, its hydrolysis product **2**, and the natural product coumarin **3** (and its hydrolysis product **4**) for which the CA inhibitory activity was first reported^6^.

Coumarins are bicyclic aromatic compounds incorporating two oxygen atoms, an endocyclic and an exocyclic one, forming thus a cyclic lactone, as shown in Figure 1 for the simplest such derivative **1**. Due to the presence of the lactone moiety, coumarins are easily hydrolysed, mainly in alkaline conditions, to the corresponding 2-hydroxy-cinnamic acid **2** – as sodium salts (usually as a *trans* isomer), which in turn, easily recyclizes (in acidic medium) with the formation of the initial coumarin and loss of a water molecule^1–5^. The chemistry of this well-known class of organic compounds was extensively reviewed and will be not discussed in detail here^1–4^.

Coumarins are widespread natural products, being present as secondary metabolites in many species of bacteria, plants, fungi, and marine organisms (such as the ascidians)^3–10^. They also possess a range of biological activities, the best-known one being that of anticoagulants^11^, although for many representatives (natural or synthetic coumarin derivatives) other pharmacological activities were reported. They include inhibition of the enzyme monoamine oxidase (MAO)^4^^,^^5^, anti-infective activity (as antibacterial, antifungal, and antiviral agents)^8^, antioxidants^2^^,^^8^, antitumor^2^^,^^4^, and anti-inflammatory action^2^^,^^4^^,^^8^, among others. In many cases (except the anticoagulant effects^11^ and MAO inhibition^4^^,^^5^) the precise mechanism of action or the real biological targets of the coumarins, active against so many different diseases/conditions, are not clearly understood. A rather different situation on the other hand is encountered for the enzyme carbonic anhydrase (CA, EC 4.2.1.1), for which the inhibitory activity of this class of compounds has been reported a decade ago^6^^,^^7^. Indeed, the first report that a natural product coumarin (compound **3**, Figure 1) acts as an inhibitor of this enzyme came from Quinn’s group^7^ who reported a high-throughput screening assay of a large library of natural products of Australasian origin and detected just one active hit, compound **3**. However, as coumarins were not among the classical chemotypes known to inhibit these enzymes^12^^,^^13^, only a subsequent detailed study^6^ demonstrated that coumarin **3** present in the Australian pant *Leionema ellipticum* (and coumarins more generally^6^^,^^10^) are indeed a new class of CA inhibitors (CAIs) with a definitely unique inhibition mechanism, the real inhibitor being, in fact, the hydrolysed coumarin derivative, **4** (Figure 1). In the following part of this review, I will examine the natural product coumarins reported to date to possess significant CA inhibitory properties and their potential use as pharmacological agents, after briefly introducing CAs and their inhibition/activation mechanisms.

## 2. Carbonic anhydrase inhibition/activation

CAs are widespread metalloenzymes in all life kingdoms, with eight diverse genetic families encoding them^12^^,^^14–19^. They catalyse the interconversion of carbon dioxide and bicarbonate, a simple but crucial reaction for a host of physiological processes in all types of organisms, since two neutral molecules, CO_2_ and water, are transformed into a weak base, bicarbonate, and H^+^ ions, a strong acid^12^^,^^14–19^. The nucleophile for achieving this transformation is a metal hydroxide species of the enzyme^12^^,^^14–19^. Thus, this reaction is fundamental for pH regulation processes in all types of cells and organisms, in healthy or diseased tissues, whereas the metabolic role of these enzymes started to be recognised only in the last period^20^^,^^21^. As a consequence, the modulation of CA activity has pharmacological applications for the management of a multitude of human diseases. Indeed, CAIs started to be used in therapy as diuretics in the 50 s^22^^,^^23^, whereas nowadays they are still used for such applications^24^, but also as anti-glaucoma agents^25^^,^^26^, antiepileptics^27^^,^^28^, and antiobesity drugs^29^^,^^30^. Furthermore, some CAIs are in various stages of clinical development for the management of metastatic, hypoxic tumours^31–34^. It should be also noted that recently, some types of CAIs showed promising activity for the management of conditions such as neuropathic pain^35^^,^^36^, cerebral ischaemia^37^, arthritis^38–40^, idiopathic intracranial hypertension^41^, and some neurodegenerative disorders^42^^,^^43^. On the other hand, the CA activators (CAAs) show pharmacological applications for memory therapy^44^ and in the modulation of emotional memory, which opens the possibility to apply them in areas with few therapeutic options at this moment, such as phobias, post-traumatic stress, generalised anxiety, and obsessive-compulsive disorders^45^^,^^46^.

**Figure 2.**
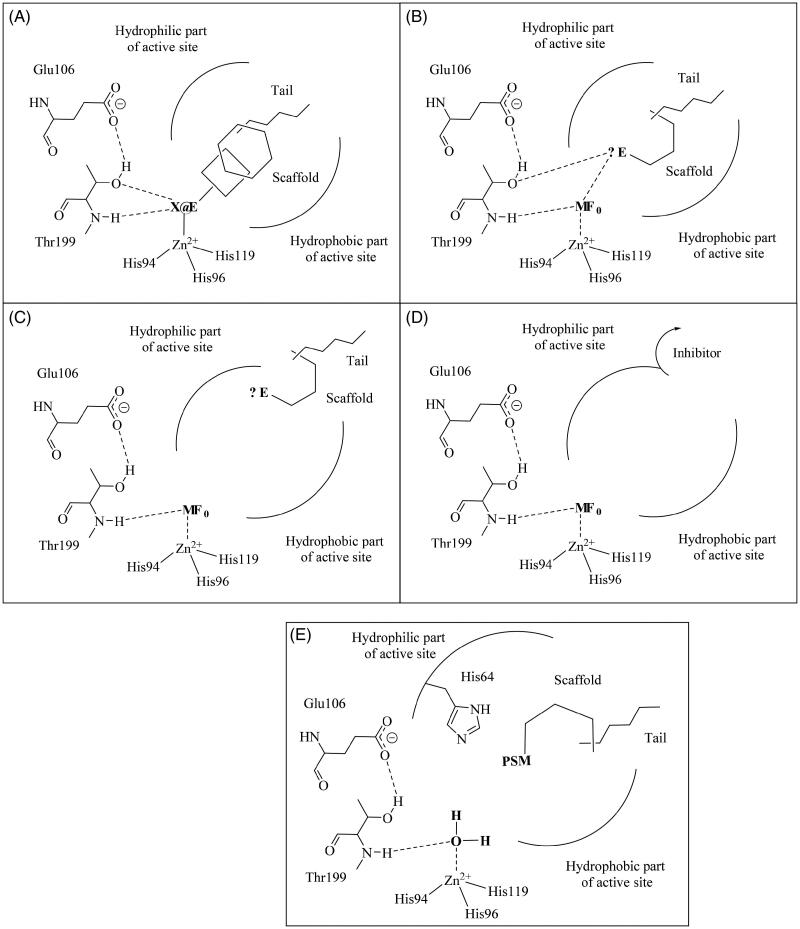
CA inhibition mechanisms (A–D) and the CA activation mechanism (E). The CA modulators of activity incorporate various scaffolds and tails in their molecule, as well as characteristic functionalities for all category: (A) the zinc binders possess a zinc-binding group (ZBG) which is coordinated to the metal ion; (B) the compounds which anchor to the zinc-coordinated water incorporate an anchoring group (AG) which hydrogen bonds with the water coordinated to the metal and the OH of Thr199; (C) AGs are also present in compounds which occlude the entrance of the active site cavity; (D) some inhibitors which bind out of the active site; (E) the activators incorporate proton-shuttling moieties (PSMs) and bind in the same active site region as the inhibitors shown at (C). Only α-class CAs are considered here (as they are the enzymes present in mammals, including humans^12^^,^^14^) although these mechanisms may be valid to other CA classes.

There are at least four CA inhibition mechanisms reported to date^12^^,^^47–50^, which together with the CA activation mechanism are schematically shown in Figure 2 for α-CAs which incorporate a catalytically crucial zinc ion at their active site^12^^,^^14^. They can be classified as follows:

1. *Inhibitors acting as zinc binders* (Figure 2(A)). These chemotypes possess a zinc-binding group (ZBG) which is coordinated to the metal ion, whereas the remaining part of their molecule interacts either with the hydrophobic, hydrophilic, or both halves of the active site^12^^,^^47–50^. Some ZBGs (sulphonamides, sulfamates, sulfamides, carboxylates, hydroxamates, benzoxaboroles, etc.) also participate in hydrogen bond interactions with two amino acid residues conserved in all α-CAs, the so-called gatekeepers, Thr199-Glu106^12^. Their scaffold can be aromatic, heterocyclic, aliphatic, or carbohydrate-based^47–50^.
2. *Inhibitors anchoring to the zinc-coordinated water molecule* (Figure 2(B)). These inhibitors incorporate an anchoring group (AG) in which hydrogen bonds with the water coordinated to the metal ion (acting as a nucleophile in the catalytic reaction), as well as with the OH of Thr199 from the gatekeeper dyad^47–50^. Phenols, polyamines, sulfocoumarins, and some other compounds inhibit CAs by this mechanism^51^^,^^52^. So, AG is usually an OH, NH_2_, or SO_3_H moieties^47–52^.
3. *Inhibitors which occlude the entrance to the active site* (Figure 2(C)). Coumarins, which will shortly be discussed in detail, are the compounds that bind in this way^6^^,^^10^^,^^53–55^, rather far away from the metal centre, at the entrance of the cavity. AG is here a COOH or phenolic OH moiety (Figure 2(C)).
4. *Inhibitors binding out of the active site* (Figure 2(D)). Only one example of such a derivative is known at the moment (2-benzylsulfonyl-benzoic acid), which binds in an adjacent hydrophobic pocket to the entrance to the active site cavity, blocking the proton shuttle of the enzyme, residue His64 in its *out* conformation, which leads to the collapse of the catalytic cycle^56^.
5. *CA activators* (Figure 2(E)). These modulators of CA activity do not abolish but enhance the catalytic efficiency of these enzymes, which are already highly effective catalysts for the CO_2_ hydration reaction^12^^,^^14^^,^^46^. The CA activators incorporate proton-shuttling moieties (PSMs) in their molecules, which most of the time are of the amino, carboxylate, or imidazole type^57–60^ and bind at the entrance of the active site cavity. Thus, the activator binding site is superimposable with the coumarin-binding site shown in Figure 2(C)^57–60^.

Nowadays, a huge number of various chemotypes are associated with the inhibition and activation of CAs, and as mentioned above some of these compounds are clinically used as drugs for the management of a variety of disorders^12^^,^^14^^,^^15^^,^^21–29^^,^^33^. However, as in humans, there are 15 CA isoforms which are rather similar from the structural viewpoint^12^^,^^14^, for a very long period it was considered impossible to obtain isoform-selective CAIs^12^. In fact, the quite large number of disorders in which inhibitors and activators of CAs show pharmacological effects is in fact due to the large number of isoforms, their diverse sub-cellular and tissue distributions, which leads to very different functions of the many CA isoforms present in various cells/tissues/organs^12^^,^^14^^,^^15^^,^^21–29^^,^^33^. Thus, the first and second generation CAIs, which all belong to the zinc binders and are sulphonamides, sulfamates, and sulfamides, are in fact still in clinical use, but they are associated with relevant side effects due to their non-isoform selective profiles and off-target inhibition^12^^,^^14^^,^^15^^,^^21–29^^,^^33^^,^^61^. Only the discovery of coumarins as CAIs^6^^,^^7^ allowed for the first time the rational drug design of isoform-selective inhibitors for all the catalytically active human(h) CA isoforms (12 of the 15 hCA isoforms possess catalytic activity, whereas hCA VIII, X, and XI are devoid of it^12^^,^^14^) As shown in Figure 2(C), these compounds, the coumarins, bind at the entrance of the hCA active site, which is the region most variable between the different isoforms^12^^,^^14^. For this reason, compounds which can bind and interact with amino acid residues in this part of the active site are generally characterised by an enhanced isoform-selectivity compared with compounds which bind deep within the active site, where a large number of the amino acid residues are conserved among the various CA isoforms^12^^,^^14^^,^^16^.

## 3. Coumarins as CAIs

**Figure 3.**
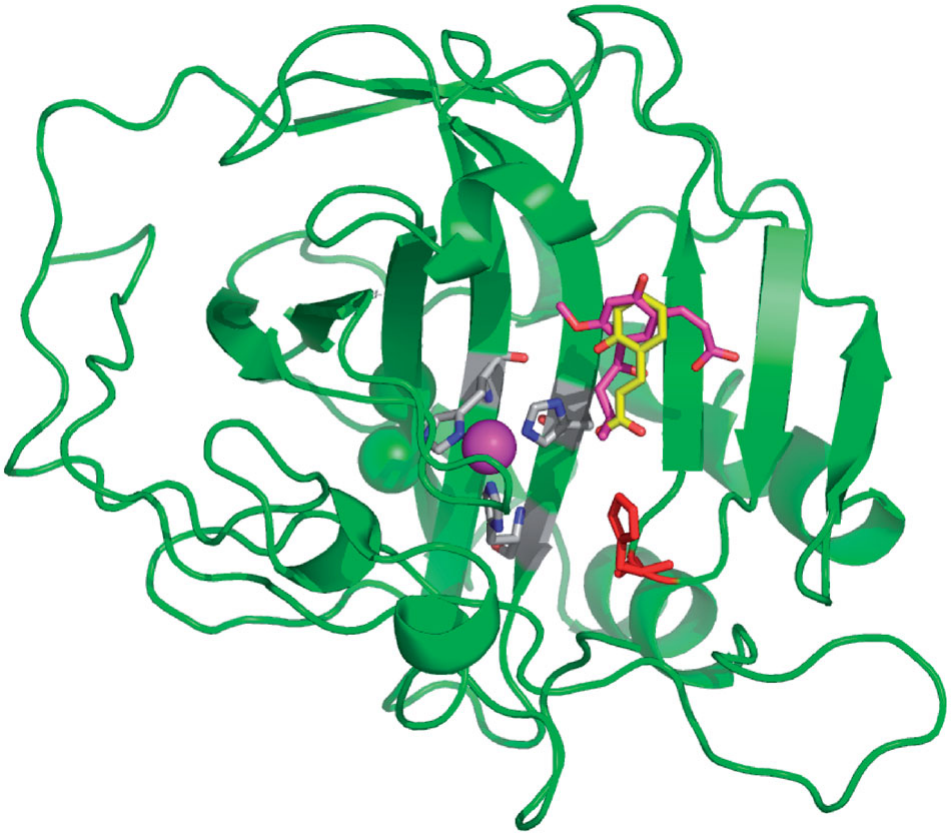
X-ray crystal structure of adducts of coumarins **1**^10^ and **3**^6^ bound to hCA II. The hydrolysis products of the two coumarins, *cis*-2-hydroxycinnamic acid **2** (in yellow) and *trans*-2-hydroxy-cinnamic acid **4** (in magenta) were observed bound wat the entrance of the CA active site. The catalytic Zn(II) ion is the central violet sphere, its three protein ligands (His94, 96 and 119, CPK colors) and the proton shuttle residue His64 (in red) are evidenced. The hCA II backbone is shown as the green ribbon, with its various α-helices, β-sheets, and loops represented in a canonical manner.

Detailed kinetic and X-ray crystallographic techniques allowed us to unravel the CA inhibition mechanism with coumarins^6^^,^^10^. The first crystal structure was that of the natural product coumarin **3**^6^ bound to human (h) isoform hCA II, followed shortly thereafter by the crystal structure of the simple coumarin **1** (Figure 1) bound to the same isoform^10^. These two X-ray crystallographic structures are superimposed in Figure 3.

Coumarins possess unique characteristics as CAIs, as determined from kinetic, mass spectrometric, and crystallographic experiments^6^^,^^10^. Unlike sulphonamides or other small molecule inhibitors, the formation of the enzyme-inhibitor complex is not a rapid process^16^, but it takes several hours^6^. Indeed, all experiments for determining the inhibition constants of these compounds are done by incubating the enzyme and the coumarins for 6 h^6^^,^^10^, whereas for other classes of CAIs the incubation period is 15 min^10^^,^^14^. This was the first indication that probably the coumarins are suicide inhibitors, which in fact was confirmed by the crystallographic experiments^6^^,^^10^. As seen from Figure 3, the coumarin ring is not present in the enzyme-inhibitor complexes formed between hCA II and the coumarins **1** and **3**, respectively. Instead, the corresponding hydrolysis products, 2-hydroxy-cinnamic acids **2** and **4** are observed bound at the entrance of the active site. This can only be achieved by the hydrolysis of the lactone ring of the coumarins through the esterase activity of hCAs, which in fact has been well documented earlier^62–64^. It is interesting to note that the unsubstituted 2-hydroxy-cinnamic acid **2** binds to the enzyme in its *trans* geometry, whereas the much bulkier derivative **4** obtained from the natural product coumarin **3** was observed in the generally less stable *cis* geometry (Figure 3). But the most relevant finding was that these compounds bind at the entrance of the active site cavity at around 8–10 Å from the zinc ion^6^^,^^10^. In this active site region, no other inhibitors were observed bound previously, confirming the uniqueness of the coumarin inhibition mechanism. Thus, coumarins are indeed suicided CAIs, which undergo hydrolysis through the esterase CA activity and bind in a region of the active site not exploited by other classes of inhibitors, but which is in fact the same binding site of the CA activators^46^^,^^59^^,^^60^. This discovery led to a large number of studies of coumarins as CAIs, both synthetic and naturally occurring derivatives.

## 4. Natural product coumarins investigated as CAIs

**Figure 4.**
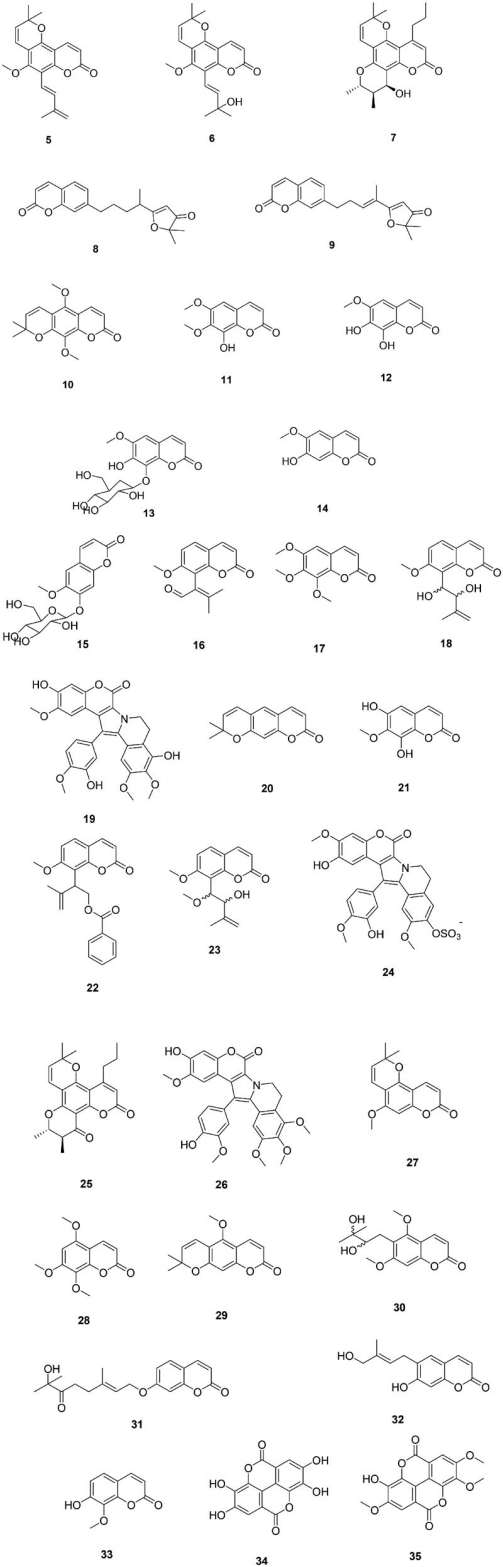
Natural product coumarins **5–35** investigated as CAIs.

The most extensive study of natural product coumarins (NPCs) acting as CAIs was reported by Davis et al.^9^ who investigated a series of such derivatives (Figure 4) isolated from plants and ascidians, of types **5–35**, for the inhibition of six hCAs (Table 1). Isoforms hCA I, II, VII, IX, XII, and XIII were investigated. The first three of them and hCA XIII are cytosolic enzymes, whereas hCA IX and XII are transmembrane, tumour-associated isoforms, present in few normal tissues^12^^,^^14^^,^^16^ and validated as antitumor drug targets^21^. The NPCs incorporate a variety of scaffolds, as shown in Figure 4, ranging from very simple coumarins with compact substituents, such as those present in **11**, **12**, **14**, **17**, **33**, etc., to rather complex/bulky substituents in various positions, or condensation with other ring systems, such as in **5–7**, **19**, **20**, **24**, **25**, **26**, **34**, **35**, etc.

**Table 1.** Inhibition data against six CA isoforms (hCA I, II, VII, IX, XII, and XIII) with coumarins **5–35**^9^.

	*K_I_* (µM)					
Compound	hCA I	hCA II	hCA VII	hCA IX	hCA XII	hCA XIII
**5**	7.66	>100	0.65	0.62	0.79	45.0
**6**	8.46	>100	8.98	0.78	0.77	29.3
**7**	9.31	50.7	8.87	0.83	0.81	21.0
**8**	59.2	63.4	9.03	0.89	0.60	27.4
**9**	9.75	>100	7.82	0.60	0.83	9.62
**10**	9.21	49.3	9.31	0.86	8.35	>100
**11**	9.89	>100	5.56	0.85	7.84	95.7
**12**	4.86	94.3	4.32	0.61	7.70	9.73
**13**	5.04	>100	3.87	0.37	7.45	9.80
**14**	10.56	>100	8.71	0.96	4.05	17.8
**15**	5.93	>100	9.11	8.72	0.78	8.43
**16**	9.11	>100	8.85	8.12	7.44	8.89
**17**	**9.7 nM**	>100	9.28	6.58	18.2	4.24
**18**	8.43	>100	29.7	3.35	8.91	4.75
**19**	6.45	>100	14.5	3.22	9.07	4.63
**20**	21.5	>100	9.18	7.51	25.7	8.36
**21**	14.0	>100	23.8	7.37	4.14	5.27
**22**	5.84	>100	>100	0.67	7.39	4.06
**23**	7.41	>100	>100	0.68	0.76	3.28
**24**	6.55	>100	78.4	3.27	1.79	4.24
**25**	8.32	>100	4.15	8.38	0.87	6.26
**26**	40.1	>100	58.3	6.33	8.51	3.70
**27**	5.60	>100	8.11	3.50	9.10	5.91
**28**	4.31	9.65	7.01	0.76	0.83	3.32
**29**	7.71	>100	6.27	0.74	0.96	3.15
**30**	2.07	>100	3.34	7.85	0.84	3.48
**31**	7.81	>100	3.69	4.03	0.70	6.10
**32**	7.52	78.9	6.92	9.75	0.77	6.35
**33**	36.4	>100	4.53	0.85	9.12	7.26
**34**	68.2	>100	8.79	79.8	8.15	4.24
**35**	44.1	>100	8.58	17.4	7.42	5.97

As seen from data of Table 1, all types of activities were observed with coumarins **5–35**, but most of the derivatives were inactive or showed poor activity against the house-keeping and most diffuse isoform, hCA II, whereas they acted as efficient, micro- or submicromolar inhibitors against the other isoforms, such as hCA I, VII, IX, XII and XIII. One compound (**17**) was a low nanomolar inhibitor of hCA I (Table 1). Generally, hCA IX and XII showed better inhibition with these NPCs compared to the other isoforms^9^. As for all other coumarins investigated to date^6^^,^^10^, nature and position (number) of substituents on the coumarin ring were the most important factors influencing the enzyme inhibitory properties of these compounds^9^.

**Figure 5.**
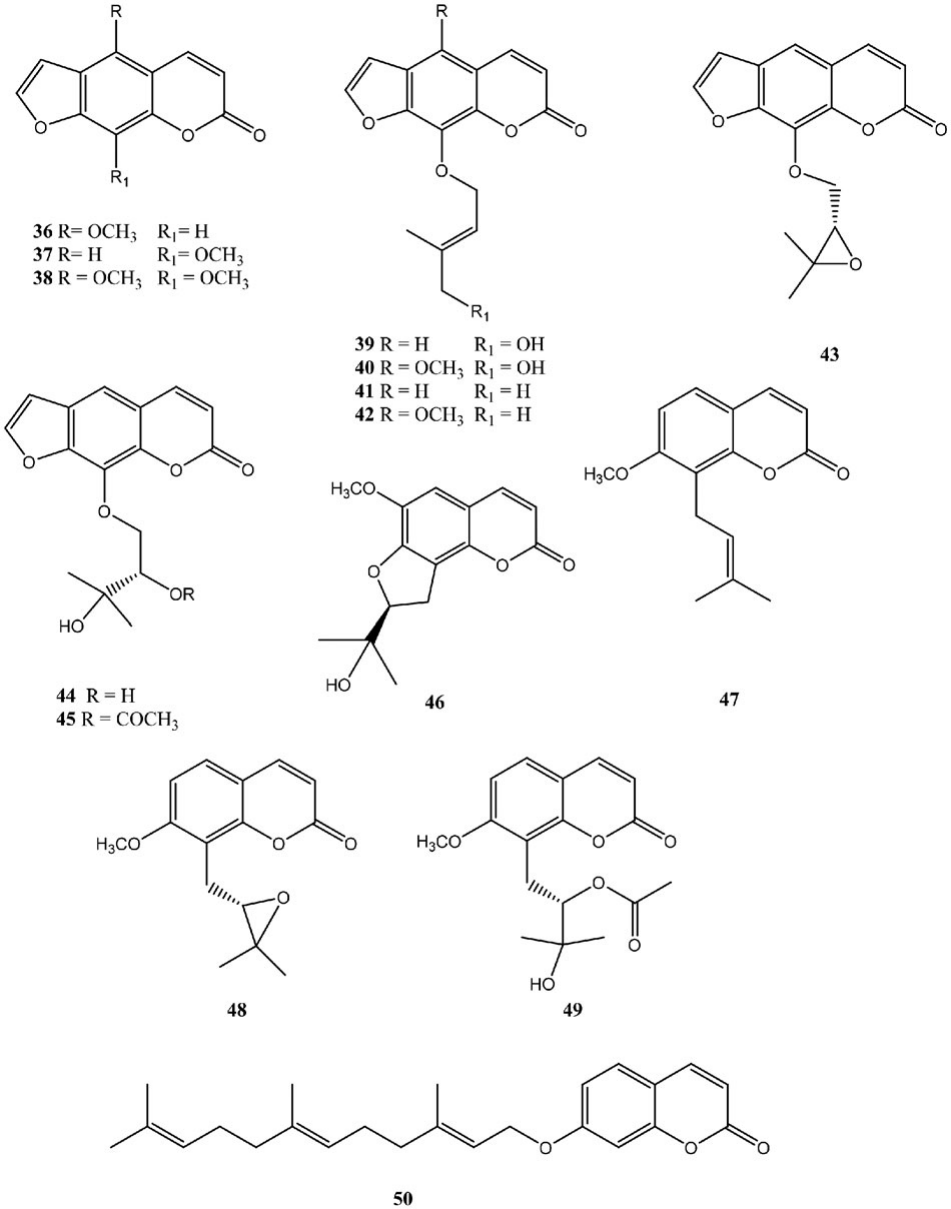
NPCs from the Sardinian plant *Magydaris pastinacea* investigated as CAIs.

Another, more recent study, by Fois et al.^65^ reported coumarins from the Sardinian plant *Magydaris pastinacea* (Figure 5) acting as efficient inhibitors of the tumour-associated isoforms hCA IX and XII whereas their activity for the cytosolic isoforms hCA I and II was found to be very weak (Table 2).

**Table 2.** hCA I, II, IX, and XII inhibition with NPCs **36**–**50**.

NPC	*K_I_* (nM)			
	hCA I	hCA II	hCA IX	hCA XII
**36**	>10,000	>10,000	1953	855.1
**37**	>10,000	>10,000	194.8	876.3
**38**	>10,000	>10,000	159.8	590.1
**39**	>10,000	>10,000	2339	550.0
**40**	>10,000	>10,000	1501	63.5
**41**	>10,000	>10,000	221.4	832.9
**42**	>10,000	>10,000	201.9	786.9
**43**	>10,000	>10,000	162.5	835.6
**44**	>10,000	>10,000	27.5	813.8
**45**	>10,000	>10,000	192.5	>10,000
**46**	>10,000	>10,000	150.9	623.0
**47**	>10,000	>10,000	2471	74.5
**48**	>10,000	>10,000	1888	>10,000
**49**	>10,000	>10,000	>10,000	290.9
**50**	>10,000	>10,000	266.4	5.8
**AAZ**	250.0	12.1	25.8	5.7

The standard sulphonamide inhibitor acetazolamide (**AAZ**) is also included.

Some of these compounds (**36–45**) are in fact variously substituted furo-coumarins (psoralens), with a tricyclic ring system incorporating the furan heterocycle, and this class of NPs is well known in nature, being present in many other plants^65^. The remaining derivatives incorporate isoprenyl-, hydrated isoprenyl- or polyprenylated moieties, also typical for many NPs^65^. It is interesting to note that all coumarins/furocoumarins **36–45** were ineffective as hCA I and II inhibitors, whereas they showed a rather good, high nanomolar inhibitory action against the tumour-associated isoforms hCA IX and XII (Table 2)^65^.

In another study, by Melis et al.^66^, several other NP coumarins and psoralen derivatives (**51–58**), this time incorporating carboxylic acid or ester moieties, were investigated as CAIs against isoforms hCA I, II, IX, and XII (Table 3). There is a continuous interest in this class of derivatives for their potential applications as anti-fungal agents^67^ or photo-activatable antitumor drugs, due to the photochemical properties of the psoralen ring system^68^.

**Table 3.** Coumarins and psoralens **51–58** and their hCA I, II, IX, and XII inhibitory action. 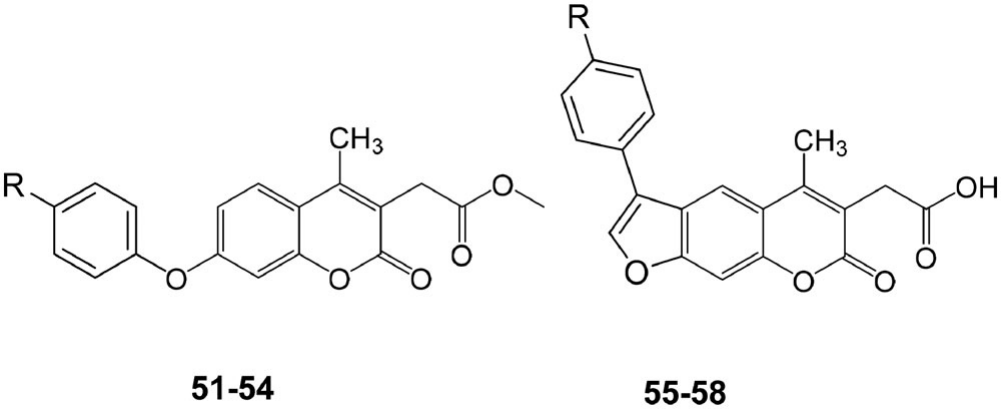

NPC	R	*K_I_* (nM)			
		hCA I	hCA II	hCA IX	hCA XII
**51**	Cl	>10,000	>10,000	23.6	446.6
**52**	Me	>10,000	>10,000	122.8	56.6
**53**	H	>10,000	>10,000	89.7	72.5
**54**	F	>10,000	>10,000	84.7	250.0
**55**	Cl	>10,000	>10,000	94.7	9.3
**56**	Me	>10,000	>10,000	23.0	9.1
**57**	H	>10,000	>10,000	17.5	9.4
**58**	F	>10,000	>10,000	17.7	7.4

As for the derivatives from *Magydaris pastinacea*, also these investigated coumarins/psoralens **51–58** showed no inhibitory action towards the cytosolic hCA I and II isoforms, which is a positive feature, as these are house-keeping enzymes in most cells/tissues, but they were effective, in some cases as low nanomolar inhibitors against the tumour associated isoforms hCA IX and XII; making them of interest for anticancer studies. In fact, already in 2011, our group demonstrated^69^ that some synthetic (not NP) coumarins incorporating glycosyl moieties show very effective *in vitro* CA IX/XII inhibitory action (with no inhibition of the off-target isoforms hCA I and II) and also a significant antitumor and antimetastatic effects in an *in vivo* animal model of hypoxic, metastatic tumours^70^.

## 5. Conclusions

Due to their unique CA inhibition mechanism, coumarins, and related compounds afforded for the first time the advent of highly isoform-selective CAIs for all human isoforms among the 12 catalytically active ones. The NP coumarins were essential for unravelling the inhibition mechanism and also for understanding in detail the structure-activity relationship for this class of enzyme inhibitors. Due to their particular inhibition mechanism, the presence of bulky moieties in the 3 position of the coumarin ring is associated with very poor or no CA inhibition. As a consequence, all the anticoagulant coumarins, which do possess very bulky moieties in position 3, do not act as CAIs, which is a positive feature both for the coumarin CAIs eventually designed for various pharmacological applications, as well as for the anticoagulants.

Apart from their highly interesting features as isoform-selective inhibitors, the discovery of coumarins as CAIs stimulated the research for the discovery of new chemotypes possessing such properties or similar inhibition mechanisms. Indeed, the monocyclic 5- and 6-ring lactones and thiolactones were reported to possess CA inhibitory effects^71^, as well as thio- and 2-thioxo-coumarins^72^, quinoline-2-ones^73^, sulfocoumarins^74^, homosulfocoumarins^75^^,^^76^ and various hetero-coumarins incorporating selenium, tellurium and other elements^77^. More recently, this type of prodrug CAI inspired also other groups to investigate this type of suicide inhibitor, such as for example aspirin, reported by McKenna’s group^78^. The huge number of synthetic studies of non-NP coumarins which have been reported in the last decade are not mentioned here, but many such compounds have been designed and showed a relevant biological activity. Overall, the very interesting moieties present in the NP coumarins make them a source of inspiration for medicinal chemists and pharmacologists in the search of new drugs with a safer profile and specific action in a variety of disorders, starting from the infective ones^79^ and ending with tumours^80^ and inflammatory diseases^81^. For the moment, only the human α-CAs were investigated for their inhibition profile with coumarins, but such enzymes are also present in other organisms, such as pathogenic bacteria, protozoans, and fungi^82–85^. Investigation of coumarin derivatives (including NPCs) against these enzymes may thus lead to interesting developments in the fight of infections produced by such pathogens.
